# Indoor Positioning in Wireless Local Area Networks with Online Path-Loss Parameter Estimation

**DOI:** 10.1155/2014/986714

**Published:** 2014-08-04

**Authors:** Luigi Bruno, Paolo Addesso, Rocco Restaino

**Affiliations:** ^1^German Aerospace Center (DLR), Institute of Communications and Navigation, P.O. Box 1116, 82230 Oberpfaffenhofen, Germany; ^2^DIEM, University of Salerno, Via Giovanni Paolo II No. 132, 84084 Fisciano, Italy

## Abstract

Location based services are gathering an even wider interest also in indoor environments and urban canyons, where satellite systems like GPS are no longer accurate. A much addressed solution for estimating the user position exploits the received signal strengths (RSS) in wireless local area networks (WLANs), which are very common nowadays. However, the performances of RSS based location systems are still unsatisfactory for many applications, due to the difficult modeling of the propagation channel, whose features are affected by severe changes. In this paper we propose a localization algorithm which takes into account the nonstationarity of the working conditions by estimating and tracking the key parameters of RSS propagation. It is based on a Sequential Monte Carlo realization of the optimal Bayesian estimation scheme, whose functioning is improved by exploiting the Rao-Blackwellization rationale. Two key statistical models for RSS characterization are deeply analyzed, by presenting effective implementations of the proposed scheme and by assessing the positioning accuracy by extensive computer experiments. Many different working conditions are analyzed by simulated data and corroborated through the validation in a real world scenario.

## 1. Introduction

Indoor positioning has been drawing remarkable interest since it is pivotal in location based services (LBS), such as visitors monitoring for security issues, automated navigation to points of interest, and customized advertising for pedestrians in malls [1, 2]. The need for local, low cost, and reliable technologies arises from the inaccurateness of satellite based navigation system indoor [3]. Wireless communication technologies, like wireless local area networks (WLANs), represent a valid alternative for their pervasive presence; moreover, the use of received signal strengths (RSSs) obtained from the beacon signals does not affect privacy issues because it does not require exchange of sensitive information.

The complexity of indoor environments has a deep impact on radio propagation, since reflection and diffraction of the radio waves on surfaces and edges make the field propagation highly random. Furthermore, since WLANs usually operate at frequencies between 2 GHz and 5 GHz, interaction with small objects causes time-variant scattering, causing diffraction and multipath contributions, which generate slow or fast fading effects, respectively [4, 5]. A further technological problem, which affects the performances of positioning algorithms, is intercalibration: different receivers have different antenna gains, thus requiring a calibration procedure that is specific for each employed device [6, 7]. The criticality of this step has attracted a relevant number of contributions of the recent devoted scientific literature [6–12].

The harshness of the indoor propagation channel modeling has endorsed the development of positioning techniques based on scene analysis (or fingerprinting), which use an empirical representation of the field emitted by the transmitting access points (APs), constituting the radio map (RM) of the environment. To this aim, an offline stage is usually performed for measuring RSS at a number of known positions (an independent localization system is required in this phase). During localization, RSS measurements collected at the unknown position are compared to the RM, allowing inferring the user location through a deterministic or probabilistic rule [13]. RADAR is the most famous fingerprinting algorithm which simply adopts RSS mean values of the RM and is shown to achieve positioning accuracy down to 2-3 meters in office buildings [14]. Although these results are very appreciable, the construction of the RM makes the algorithm hardly scalable with the size of the building and, above all, variability of radio propagation should be accounted in order to make the algorithm robust. In [6] the RM is periodically corrected under the arbitrary assumption that the change is uniform across the area. A more flexible system is proposed in [15] which makes use of model trees to adapt the RM online by using RSS measurements at some reference points and without assuming explicit transformation functions. More recently, [11, 16] propose the use of projections techniques to extract features from the RM, which can be more easily updated during the online stage. Focusing on the related problem of intercalibration in [9] develops a solution for addressing the incoherence of the RM with the current operating conditions based on a transformation function, whose training online causes a transient in the algorithm performance (1-2 minutes in the proposed real scenarios).

Although the cited techniques can alleviate the variability issue at the cost of a moderate increase of complexity, the need for an on-site training of the RM still represents the principal drawback of fingerprinting approaches. The development of methods exploiting a theoretical propagation model constitutes the unique possibility of avoiding this demanding step. In this case the key phase is represented by an accurate statistical characterization of the RSS. The* path-loss model*, based on Friis formula, is a very addressed representation for radio propagation and consists in an additive model (in decibel) composed of a deterministic part, accounting for the mean intensity and a zero-mean random term.

The first factor is completely specified by two parameters: the transmitted power, which depends also on the antenna gains, and the path-loss exponent, which describes the decay of signal intensity with distance [17]. The sensitivity of positioning algorithms to errors on path-loss parameters and different solutions for the setting of this crucial quantities has been explored in several papers, for example, in [18], where an empirical study based on RSS measurements in the IEEE 802.11.b network is proposed. Some authors focus on the sole path-loss exponent, with the aim of optimizing least squares position estimation methods [8, 19] or of mitigating the impact of its uncertainty in the spring-relaxation algorithm [20].

The second term of the path-loss model characterizes the random nature of the RSS. Accordingly, it is commonly used to describe the principal corruption effects due to the indoor propagation channel disturbances and in particular the fading effects due to diffraction and reflection phenomena. A widespread model consists in employing a Gaussian distribution for describing the additive random contribution to the RSS in dB. Actually this model, whose success is especially due to its mathematical tractability, is particularly suited for describing the received signal intensity in the presence of slow fading, which corresponds to a Lognormal distribution of the RSS in linear measure units [4]. On the other side, the Gaussian hypothesis is often unrealistic, as it happens, for example, when fast fading effects are present [4]. According to this observation, some authors have dropped out the parametric functional description of the statistical model, resorting to an approach based on a demanding kernel-based density estimation method [21].

In this paper we develop a sequential Bayesian localization algorithm, aimed at reducing the effect of the inaccurate propagation model knowledge, which commonly affects the indoor positioning problem. The Bayesian scheme constitutes the recursive implementation of the maximum a posteriori probability approach [22] for the estimation of the whole mobile user trajectory. Exploiting the correlation between successive positions has been proven useful also for fingerprinting approaches [23], but the Bayesian scheme represents the most used framework for encompassing this information [24]. The objective of this work is to improve the applicability of this approach by incorporating an estimation phase, which is able to adapt the algorithm to different working scenarios, without requiring a preliminary training phase. This last goal distinguishes the method described in this paper from similar contributions that aim at jointly estimating the user position and the path-loss parameters [25].

More in detail, the proposed algorithm allows to keep on tracking parameters online, by simultaneously estimating the user's trajectory and the path-loss parameters for all APs. The method is based on a particle filter implementation [26], whose suitability for indoor localization was already shown in [27]. In a previous study, we have developed and tested a simple joint Bayesian algorithm, in which the unknown parameters were added to the state space and sampled from a fictitious Gaussian process [28]. In this paper we develop a more advanced localization algorithm based on the Rao-Blackwellized Particle Filter [29]. In this paper we only deal with one parameter of the transmitted power, this way accounting for time-varying obstacles and intercalibration, while the path-loss exponent is approximated by the free space value. This fits several empirical studies, which evidence the appropriateness of affine transformations for modeling the intensities differences between the various devices, and, more specifically, the similarity of the experienced power decay coefficients [7, 30]. Indeed, the latter parameter is mainly influenced by the propagation characteristics of the specific environment, thus resulting essentially independent of the user equipment.

A second main contribution of this paper concerns the extension of the proposed Bayesian algorithm to non-Gaussian statistical model. More specifically, a general approximate approach for implementing the Rao-Blackwellization scheme is presented. This generalization is applied to the crucial case of fast fading, for which the statistical model based on the Rice (or Nakagami-n) distribution is employed [4].

The paper is composed of the following. In Section 2 we detail the state space dynamic system employed for describing the user motion and the observed signal, with particular focus on the statistical characterization of the RSS. In Section 3 the Bayesian approach to the simultaneous estimation of state and parameters is presented, while the computer simulations, performed to analyze the performance of the proposed scheme for adaptive indoor positioning, are shown in Section 4. In Section 5 the results are validated on a real scenario (an indoor parking lot). Final remarks and further lines of research arising from this study are reported in Section 6.

## 2. State and Observation Models

The algorithm proposed in this paper estimates the location of a mobile user based on the RSS measurements. More specifically, we use a Bayesian sequential approach, which tracks the user during walk by using several scans of RSS. A crucial step of Bayesian approaches is the choice of suitable statistical models for both mobile user kinematics and received signals, which are required to yield an accurate description within an affordable mathematical framework.

In this work we ignore the vertical coordinate of the user position, which is thus encoded in the two-dimensional vector ***θ*** ∈ *R* ^2^. The movement is described according to a discrete linear nearly constant velocity model (NCVM), sampled at the time instants *kτ* [31]

(1) $$\begin{array}{c} x_{k+1}=Fx_{k}+v_{k},\;k=0,1,2,\ldots , \end{array}$$

in which the state **x**_*k*_ is the 4-dimensional vector composed of the user's position and velocity

(2) $$\begin{array}{c} x_{k}={\left[\theta_{k}^{T},{\dot{\theta }}_{k}^{T}\right]}^{T}, \end{array}$$

where the superscript *T* indicates the transposition operator and **v**_*k*_ are the samples of a zero-mean white process, henceforth supposed Gaussian. In (1) the 4 × 4 matrix *F* is defined like

(3) $$\begin{array}{c} F=\left(\begin{array}{cc} 1 & \tau \\ 0 & 1 \end{array}\right)⊗I_{2}, \end{array}$$

having for simplicity introduced the identity matrix **I**_2_ of order 2 and the Kronecker product ⊗. The covariance matrix *Q* of the noise **v**_*k*_ is

(4) $$\begin{array}{c} Q=E\left[v_{k}v_{k}^{\prime }\right]=\sigma_{v}^{2}\left(\begin{array}{cc} \frac{1}{3}\tau^{3} & \frac{1}{2}\tau^{2}\\ \frac{1}{2}\tau^{2} & \tau \end{array}\right)⊗I_{2}, \end{array}$$

where *σ*_*v*_^2^ is the noise variance and multiplies all entries. In other terms, the velocity changes over a sampling period *τ* are of the order of

(5) $$\begin{array}{c} \sqrt{Q_{22}}=\sigma_{v}\sqrt{\tau }. \end{array}$$

In particular, the key assumption of the NCVM is that the expected velocity variations are much smaller than the actual velocity. Finally, at *k* = 0, we assign a known prior distribution *p*_**x**_(**x**_0_) to the state.

The mobile user device collects signals transmitted by *N*_AP_ APs, which are deployed in the environment in known positions ***θ***_*j*_^AP^ ∈ *A*, *j* = 1,…, *N*_AP_. Several statistical models are available in the technical literature for describing the amplitudes *r*_*j*,*k*_ of the radio signal emitted by the *j*th AP and received by the user at instant *k* [32]. We selected two credited models that are able to describe the most common signal degradations. In the case of slow fading, the conditional probability density function (pdf) of *r*_*j*,*k*_ is well described by a Lognormal distribution [4]

(6) $$\begin{array}{c} r_{j,k}\sim p_{L}\left(r\right)=\frac{1}{\sqrt{2\pi }\sigma_{j,k}r}\exp\left(-\frac{{\left(\ln r-\mu_{j,k}\right)}^{2}}{2\sigma_{j,k}^{2}}\right), \end{array}$$

where *μ*_*j*,*k*_ and *σ*_*j*,*k*_ are the pdf parameters, dependent on the distance between user and AP; instead, fast fading is better fitted by the Rice (or Nakagami-n) distribution [4]

(7) rj,k~pR(r)=2(1+Kf)rΩj,kexp⁡(−Kf−(Kf+1)r2Ωj,k)·I0(2rKf(Kf+1)Ωj,k), r≥0,

whose parameters are *K*_*f*_ ≥ 0 and *Ω*_*j*,*k*_ = *E*[*r* ^2^] and *I*_0_(·) is the zero-th order modified Bessel function of the first type. In detail, *K*_*f*_ is related to the signal-to-noise ratio of the received signal and is reported to assume values in the range *K*_*f*_ ∈ [0,20] [33].

In both cases, by expressing the amplitudes in dBm,

(8) $$\begin{array}{c} y_{j,k}=20\;\log_{10}r_{j,k}, \end{array}$$

the noise becomes an additive component. In the slow fading case the measurements in dBm follow a Gaussian pdf, with mean and variance

(9) $$\begin{array}{c} \text{E}\left[y_{j,k}\right]=\kappa \mu_{j,k}, \end{array}$$

(10) $$\begin{array}{c} \text{VAR}\left[y_{j,k}\right]=\kappa^{2}\sigma_{j,k}^{2}, \end{array}$$

where *κ* = 20/ln⁡(10).

In the case of fast fading, the conditional pdf of the RSS in dBm is

(11) yj,k~pR(y) =2(1+Kf)κ  ×exp⁡(2(y−ΩdB,j,k)κ−Kf−(Kf+1)exp⁡(2(y−ΩdB,j,k)κ))  ·I0(2Kf(Kf+1)exp⁡(y−ΩdB,j,kκ)),

where *Ω*_*dB*,*j*,*k*_ = (*κ*/2)ln⁡(*Ω*_*j*,*k*_) is a shift parameter and affects only the expectation

(12) $$\begin{array}{c} \text{E}\left[y_{j,k}\right]=\Omega_{dB,j,k}-e\left(K_{f}\right), \end{array}$$

(13) $$\begin{array}{c} \text{VAR}\left[y_{j,k}\right]=v\left(K_{f}\right). \end{array}$$

**Figure 1:**
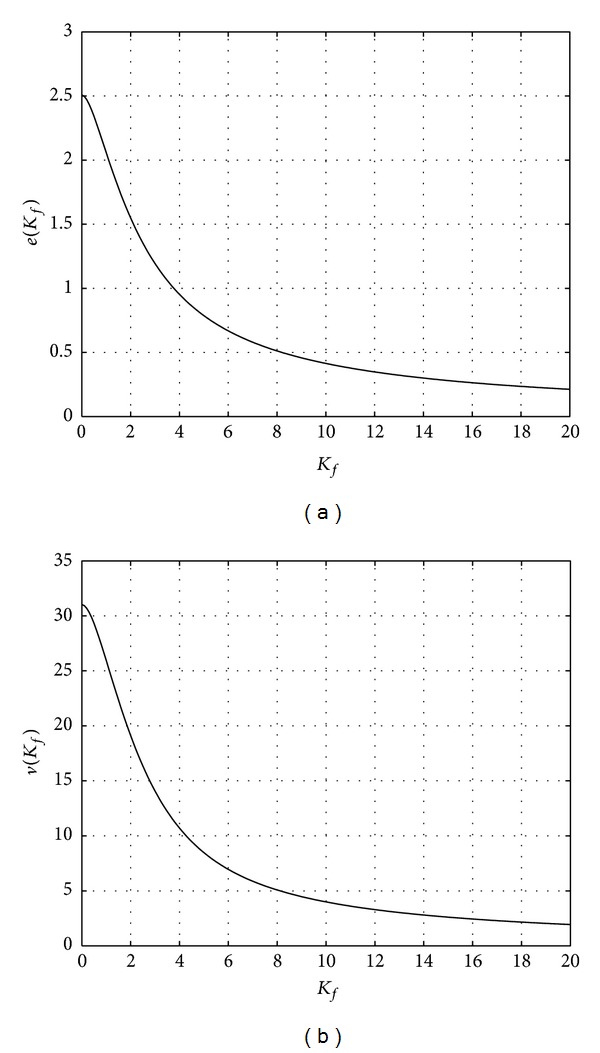
Plot of the functions *e*(*K*_*f*_) (a) and *v*(*K*_*f*_) (b) versus *K*_*f*_.

The functions *e*(*K*_*f*_) and *v*(*K*_*f*_) can be numerically evaluated and they are depicted in Figure 1 in a typical range for *K*_*f*_.

These considerations motivate the use of the additive model for the observations, since for both slow and fast fading cases the RSS measurement can be written as

(14) $$\begin{array}{c} y_{j,k}=\text{E}\left[y_{j,k}\right]+n_{j,k}, \end{array}$$

where E[*y*_*j*,*k*_] is given by either (9) or (12) and *n*_*j*,*k*_ are the zero-mean observation noises, which are supposed to be independent among the APs. In the slow fading case *n*_*j*,*k*_ is a zero-mean Gaussian variable with variance given by (10), while in the presence of fast fading *n*_*j*,*k*_ is distributed like in (11), but with zero mean, as obtained by setting *Ω*_*dB*,*j*,*k*_ = *e*(*K*_*f*_). The expected RSS value E[*y*_*j*,*k*_] models the average attenuation experienced by the strength of the signal emitted by *j*th AP at a given distance *d*_*j*_ = ||***θ***_*k*_ − ***θ***_*j*_^AP^||. Its value in dBm is commonly described through the path-loss model [17]

(15) $$\begin{array}{c} \text{E}\left[y_{j,k}\right]\hat{=}h_{j}-20\alpha_{j}\;\log_{10}\left(\frac{d_{j}}{d_{0}}\right), \end{array}$$

where *d*_0_ is a reference distance and *h*_*j*_ and *α*_*j*_ are static parameters denoting the RSS value at distance *d*_0_ and the path-loss decay exponent, respectively.

By defining the observation vector **y**_*k*_ = [*y*_1,*k*_,…, *y*_1,*N*_AP__]^*T*^, the observation noise vector **n**_*k*_ = [*n*_1,*k*_,…, *n*_1,*N*_AP__]^*T*^ and the nonlinear functions **g**(**x**_*k*_) = −20*α*_*j*_ log⁡_10_(||***θ***_*k*_ − ***θ***_*j*_^AP^||/*d*_0_), (15) can be put in a vector form

(16) $$\begin{array}{c} y_{k}=g\left(x_{k}\right)+h+n_{k}, \end{array}$$

which evidences the linear dependence of observations **y**_*k*_ on the parameters **h** and the nonlinear dependence of **y**_*k*_ on the state **x**_*k*_.

## 3. Online Sequential Bayesian Estimation of State and Parameters

We assume an incomplete knowledge of the path-loss model (15). More in detail, the parameters *h*_*j*_,  *j* = 1,…, *N*_AP_, in (15) are unknown, while the decay exponents *α*_*j*_ = *α* are set to a fixed value. This formalization fits realistic situations in which the AP's transmitted powers or, more frequently, the sensitivity of the receiving antennas is unavailable.

The localization of the mobile user is here recast within the sequential Bayesian framework, which aims at estimating its whole trajectory by means of the observations acquired at successive instants. Besides motion and observation models, we provide a fictitious probabilistic model to the parameter vector **h**, based on the identity transition matrix with the addition of noise [34]

(17) $$\begin{array}{c} h_{k+1}=h_{k}+r_{k}, \end{array}$$

in which **r**_*k*_ is assumed to be a Gaussian white noise with zero mean and a suitable covariance matrix *R*_*k*_^*r*^.

Summing up, the dynamics of the faced localization problem can be resumed by the dynamic system

(18) $$\begin{array}{c} x_{k+1}=Fx_{k}+v_{k}, \end{array}$$

(19) $$\begin{array}{c} h_{k+1}=h_{k}+r_{k}, \end{array}$$

(20) $$\begin{array}{c} y_{k}=g\left(x_{k}\right)+h_{k}+n_{k}, \end{array}$$

with priors *p*_0_(**x**_*k*_) and *p*_0_(**h**) at time 0.

As estimator of the mobile user trajectory we use the maximum a posteriori probability (MAP) estimate, given the available observations. In other terms, it consists in maximizing, at each instant *k*, the posterior pdf of the user's trajectory **x**_0:*k*_ = [**x**_0_,…, **x**_*k*_], given the RSS measurements **y**_1:*k*_ = [**y**_1_,…, **y**_*k*_], namely, in finding

(21) $$\begin{array}{c} {\hat{x}}_{0:k}=\arg\underset{x_{0:k}}{\max}p\left(x_{0:k}\;|\;y_{1:k}\right). \end{array}$$

The calculation of the state posterior pdf at *k*, given the observed data

(22) $$\begin{array}{c} p\left(x_{0:k}\;|\;y_{1:k}\right), \end{array}$$

can be obtained through the recursive factorization:

(23) p(x0:k ∣ y1:k)∝p(yk ∣ x0:k,y1:k−1)  ·p(xkx0:k−1,y1:k−1)·p(x0:k−1y1:k−1),

which is a straightforward consequence of the Bayes theorem.

The term

(24) $$\begin{array}{c} p\left(x_{k}\;|\;x_{0:k-1},y_{1:k-1}\right)=p\left(x_{k}\;|\;x_{k-1}\right) \end{array}$$

is completely specified by the model used to derive (18), which drops the dependence of **x**_*k*_ on **x**_0:*k*−2_ and **y**_1:*k*−1_, given **x**_*k*−1_; the last right term of (23) is the posterior pdf at instant *k* − 1.

On the other side, the evaluation of the first term of (23), corresponding to the RSS likelihood function, requires the marginalization over **h**:

(25) p(yk ∣ x0:k,y1:k−1) =∫p(yk ∣ xk,hk)p(hk ∣ x0:k,y1:k−1)dhk =∫p(yk ∣ xk,hk)p(h ∣ x0:k−1,y1:k−1)dhk.

In the last line we dropped the dependence of the parameter pdf on the current state since the corresponding measurement is missing. Evaluation of the integral (25) constitutes the key point of the adopted Bayesian procedure and highlights the dependence of the MAP user trajectory on the parameters distribution.

### 3.1. Existing Approaches

Dual and joint estimation algorithms constitute the most diffuse approaches to Bayesian estimation in the presence of unknown parameters. The first one consists in running two interacting concurrent algorithms, one devoted to the state estimation and another devoted to the parameters [35]. Instead, joint estimation is performed by constructing a single augmented state vector including both the kinematic quantities, namely, the position and the velocity of the mobile user, and the unknown parameters [36].

Classical Bayesian approaches for state and parameters estimation rely upon the use of Kalman filters (KFs), which are optimal for linear dynamical systems corrupted by Gaussian noise. Extended KFs (EKFs), achieved after the linearization of the equations, are a suitable solution also in the presence of nonlinear models, for both dual and joint estimation methods [34].

More accurate implementations of Bayesian algorithm for general dynamic equations are constituted by Monte Carlo schemes, which are commonly referred to as* particle filters* [26]. In this approach an empirical approximation of the posterior pdf, consisting in a summation of delta measures centered at a finite set of support points (or particle), is employed to simplify the computation of the Bayesian procedure recursions. Application of particle filtering to the joint estimation of position and propagation parameters of a mobile user connected to a WLAN has been tested in a previous contribution by the authors [28]. In this paper the sequential importance sampling with resampling (SIR) scheme [37] has been employed, underlying the capabilities of the methods, but evidencing, at the same time, its drawbacks. The most critical issue is surely related to the augmentation of the state space dimensionality; this is due to the addition of the parameters to the vector of estimating quantities. This implies the exponential growth of the particles number, required to preserve an adequate particle density within the state space.

### 3.2. Rao-Blackwellized Particle Filter

In this paper we attain the solution of the Bayesian problem through a different approach, which exploits the Rao-Blackwell Theorem to reduce the state estimation error by means of the parameter marginalization [29]. More in detail, the* Rao-Blackwellized Particle Filter* (*RBPF*) consists in applying the Monte Carlo approximations only for the state estimation and in deriving the parameter pdf through analytical procedures, instead. This is done to avoid including the parameters in the state space, which, therefore, keeps a constant dimensionality. Accordingly, the main hypothesis required for its utilization consists in the availability of a deterministic algorithm to recursively compute the parameter conditional pdf. A noticeable case is represented by parameters evolving, given the state, according to a* conditionally linear Gaussian* (*CLG*) system.

According to the Monte Carlo rationale, the user's state posterior pdf at *k* is written as

(26) $$\begin{array}{c} p\left(x_{k}\;|\;y_{1:k-1}\right)=\overset{N}{\underset{i=1}{\sum }}w_{i}\delta \left(x_{k}-x_{k}^{i}\right) \end{array}$$

in which *δ*(**x**_*k*_ − **x**_*k*_^*i*^) denotes the delta measure centered at the support point **x**_*k*_^*i*^ and *w*_*i*_ is the corresponding weight. In the SIR scheme adopted in this work, the *i*th particle **x**_*k*_^*i*^ is obtained by sampling the state space according to the predictive pdf:

(27) $$\begin{array}{c} x_{k}^{i}\sim p\left(x_{k}\;|\;x_{0:k-1}^{i}y_{1:k-1}\right)=p\left(x_{k}\;|\;x_{k-1}^{i}\right), \end{array}$$

which is replaced, at the initial time *k* = 0, by the prior

(28) $$\begin{array}{c} x_{0}^{i}\sim p\left(x_{0}\right). \end{array}$$

For *k* > 1, the particle weights are obtained in a recursive way by following the factorization illustrated in (23), namely, as

(29) $$\begin{array}{c} w_{k}^{i}=w_{k-1}^{i}\cdot p\left(y_{k}\;|\;x_{0:k}^{i},y_{1:k-1}\right), \end{array}$$

(30) =wk−1i·∫p(yk ∣ xki,h)×p(h ∣ x0:k−1i,y1:k−1)dh,

whereas for *k* = 0 the initial weights are uniformly set to *w*_0_^*i*^ = *N* ^−1^, ∀*i* = 1,…, *N*. Therefore, in order to completely specify the RBPF algorithm, we need to compute the parameters density function, conditioned on the state trajectory sample, **x**_0:*k*_^*i*^, and on data **y**_1:*k*_:

(31) $$\begin{array}{c} p\left(h\;|\;x_{0:k}^{i},y_{1:k}\right). \end{array}$$

In the following sections we detail two approaches for calculating *p*(**h**∣**x**_0:*k*_^*i*^, **y**_1:*k*_), with reference to the observation models presented in Section 2. The former represents an efficient and exact implementation, which fits the CLG model of parameters, as it is the case of Lognormally distributed noise; the latter is a very general method that constitutes an approximated solution exploitable for all nonlinear non-Gaussian (NLNG) models.

### 3.3. Lognormal Fading: Continuous Model for the Parameter

If the RSS likelihood function is assumed to be Lognormal or, equivalently, data in dBm follow a Gaussian distribution, the parameter pdf (31) can be computed by means of the Kalman filter (KF) [34]. In particular, by starting from a Gaussian prior also for the parameter vector, the integrand function in (25) is always the product of two Gaussian distributions. The result is a Gaussian density, except for a normalization constant *c*, 0 < *c* < 1,

(32) $$\begin{array}{c} p\left(y_{k}\;|\;h,x_{k}^{i}\right)p\left(h\;|\;x_{0:k-1}^{i},y_{1:k-1}\right)=cs\left(h\right), \end{array}$$

where the Gaussian pdf has been denoted by *s*(·) and its mean and variance can be easily obtained. Indeed, one can use the following result: if *s*_*i*_(**x**) ~ *N*(**m**_*i*_, Σ_*i*_), *i* = 1,2, the function *s*(**x**) = *s*_1_(**x**) · *s*_2_(**x**) is proportional to a multivariate Gaussian pdf with mean and covariance matrix **m** = (Σ_1_^−1^+Σ_2_^−1^)^−1^(Σ_1_^−1^ **m**_1_ + Σ_2_^−1^ **m**_2_), Σ = (Σ_1_^−1^+Σ_2_^−1^)^−1^. Note that the first factor on the left part of (32) is a normal pdf w.r.t. **y**_*k*_: here we further exploit the Gaussianity of **h** which can be easily derived by solving 13 for **h**, yielding

(33) $$\begin{array}{c} h=g\left(x_{k}\right)+y_{k}+n_{k}. \end{array}$$

By using (32) in the integral (30) defining the particle weight, we find

(34) $$\begin{array}{c} w_{k}^{i}\propto w_{k-1}^{i}\cdot c, \end{array}$$

with *c* being on turn the ratio

(35) $$\begin{array}{c} c=\frac{p\left(y_{k}\;|\;h,x_{k}^{i}\right)p\left(h\;|\;x_{0:k-1}^{i},y_{1:k-1}\right)}{s\left(h\right)}, \end{array}$$

which can be calculated at an arbitrary value of the variable **h**, for example, at its expected value. This algorithm will be referred to as RBPF-KF in the following.

### 3.4. General Case: Discrete Model for the Parameter

If the RSS distribution is not Gaussian, as in the fast fading case, we need another method to evaluate the integral of (25). Unfortunately numerical techniques often represent a bottleneck from a computational point of view and, therefore, we use a grid-based approach, that is computationally suitable even for nonlinear and non-Gaussian (NLNG) models. In detail, we decompose the range of variation of the *j*th component *h*_*j*,*k*_ of the vector parameter **h**_*k*_ into a finite number *N*_*c*_ of disjoint cells {*H*_*c*_}_*c*=1,…,*N*_*c*__ and quantize the values within each cell to its mean value, say ${\bar{h}}_{c}$. In different words, the parameter vector is approximated by a discrete random process ${\tilde{h}}_{k}$, whose independent components ${\tilde{h}}_{j,k}$ assume values in the set ${\left\{{\bar{p}}_{c}\right\}}_{c=1,\ldots ,N_{c}}$ and admit a probability mass function given by

(36) $$\begin{array}{c} \text{Pr}\left\{{\tilde{h}}_{j,k}={\bar{h}}_{c}\right\}=\text{Pr}\left\{h_{j,k}\in H_{c}\right\}=\int_{H_{c}}p\left(h_{j,k}\right)dh_{j,k}. \end{array}$$

By resorting again to the factorization of the posterior pdf reported in (23), we address the RBPF algorithm, but we compute differently the terms concerning the parameters. In particular, in such hypotheses, the parameter distribution corresponding to the *i*th particle is given by the pmf defined in (36). Recursive computation of the above distribution can be performed by means of the* Approximated Grid-Based* (*AGB*) algorithm presented in [37], which is the counterpart of the KF in a discrete state space. We denote this algorithm by RBPF-AGB.

## 4. Computer Experiments

**Figure 2:**
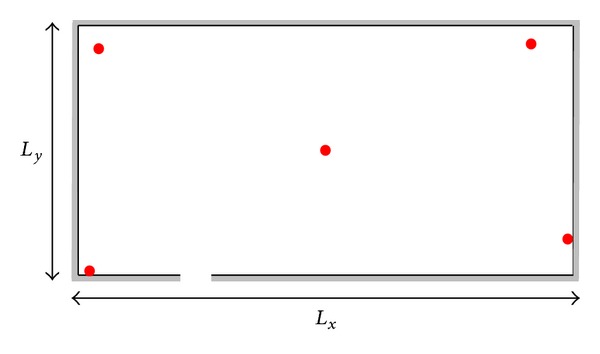
Testbed adopted in the simulations; in the figure *L*_*x*_ = 40 m and *L*_*y*_ = 20 m and the APs are in the positions denoted by red circles.

Several simulations were designed in order to analyze the performance of our proposals. We have chosen to separately evaluate the effects of fast and slow fading to avoid combined effects which would be difficult to discriminate. The synthetic testbed, represented in Figure 2, is composed of a 40 × 20 m open area where 5 APs, denoted by red circles, periodically emit beacon signals.

A user walks according to the model described in Section 2 with *σ*_*v*_ = 0.1 m/s^2^ and *τ* = 1 s. The initial state **x**_0_ is drawn from a multivariate Gaussian (MG) prior distribution with diagonal covariance matrix, whose nonzero terms are set to 1 for the positions (*σ*_*θ*1_^2^ and *σ*_*θ*2_^2^) and 0.1 for the velocities ($\sigma_{\dot{\theta }1}^{2}$ and $\sigma_{\dot{\theta }2}^{2}$). The mean RSS is given by the path-loss model described by (15), in which the free space value *α* = 2 is assumed for all the APs. In particular we draw the starting value of **h** from a MG distribution with known mean **h**_0_ and diagonal covariance matrix with elements *σ*_*h*_^2^; in some simulation settings a stepwise variation of some component of **h** is also impressed. Finally, all results are averaged over a series of independent experiments and are presented in terms of a numerical evaluation of the positioning RMSE.

### 4.1. Slow Fading (RBPF-KF)

To test the slow fading effects, measurements in dB are drawn according to a multivariate distribution whose components are independent Gaussian random processes with means given by (15) and a common fixed variance *σ*_*h*_.

**Figure 3:**
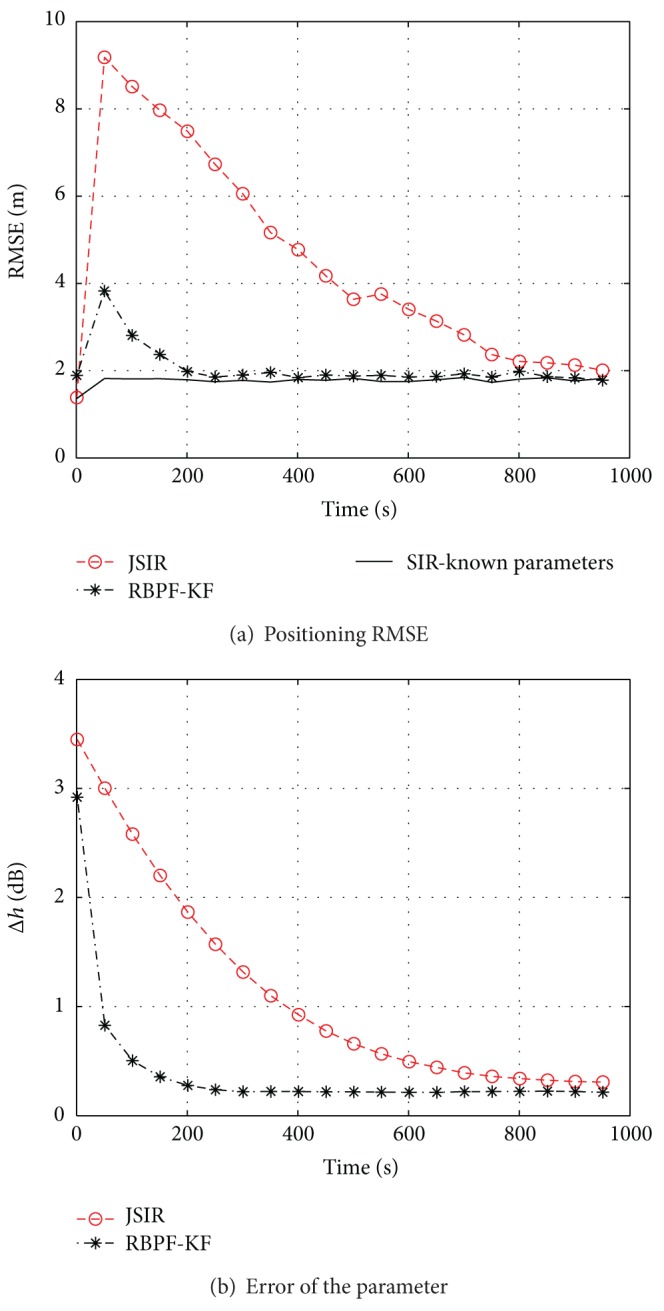
Slow fading effect evaluated by means of computer experiments based on the testbed of Figure 2. JSIR and RBPF-KF are applied with *σ*_*h*_^2^ = 9, *σ*_*y*_^2^ = 5, and *N*_*p*_ = 1000 particles; we show (a) the positioning RMSE and (b) estimation error Δ*h*_*k*_ of the parameters averaged over all APs.

Thus, we employ the RBPF-KF and compare its performances with the JSIR approach presented in [28]. The first test is carried out by setting *σ*_*h*_^2^ = 9 and, as a reference, we also draw the corresponding performance obtained by the clairvoyant SIR algorithm that is fed up with the true values of the reference power **h**. All algorithms are applied with 1000 particles and their RMSEs, calculated only on the user position, are plotted against time in Figure 3(a). The initial RMSE value is related to the covariance matrix of the state prior; namely,

(37) $$\begin{array}{c} \sqrt{E\left[{||{\hat{\theta }}_{0}-\theta_{t}||}^{2}\right]}=\sqrt{\sigma_{\theta 1}^{2}+\sigma_{\theta 2}^{2}}=\sqrt{2}; \end{array}$$

then, both adaptive algorithms are characterized by a transient during which the parameters are estimated; after this phase they attain the same performance shown by the clairvoyant algorithm. The differences between JSIR and RBPF-KF lay in the amplitude of the RMSE overshoot and in the speed of convergence; in both cases relevant benefits are achieved by RBPF. This is a direct consequence of the algorithms adaptivity: as it is shown in Figure 3(b), the error, averaged over all APs, of the estimated reference power $\hat{h}$,

(38) $$\begin{array}{c} \Delta h_{k}=\frac{1}{N_{\text{AP}}}\overset{N_{\text{AP}}}{\underset{j=1}{\sum }}\left|h_{j,k},-{\hat{h}}_{j,k}\right|, \end{array}$$

is rapidly torn down in the RBPF case to a steady state value.

**Figure 4:**
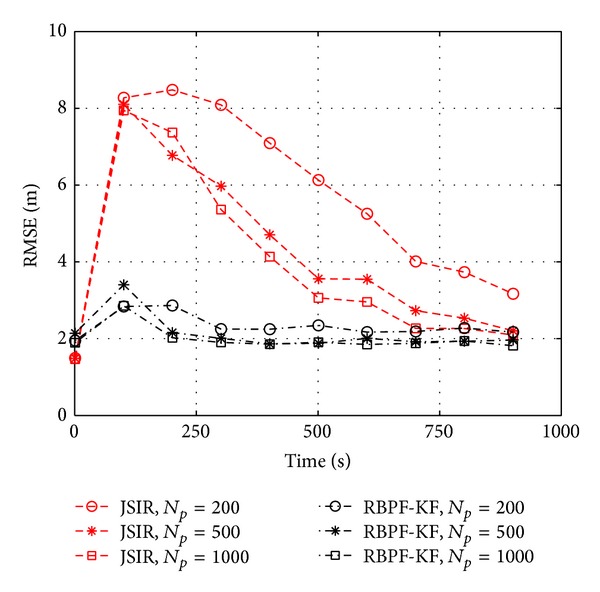
Positioning RMSE (both JSIR and RBPF-KF) related to computer experiments concerning slow fading in the setup of Figure 2 with variable number of particles in the range *N*_*p*_ = 200 ÷ 1000; here, *σ*_*h*_^2^ = 9, *σ*_*y*_^2^ = 5.

Let us dig deeper into the algorithms evaluation. In Figure 4 we show the results of our algorithms applied in the same conditions as in Figure 3 but with a variable number of particles in the range *N*_*p*_ = 200 ÷ 1000. Even 200 particles are sufficient for RBPF in order to overcome JSIR applied with as many as 1000 particles.

**Figure 5:**
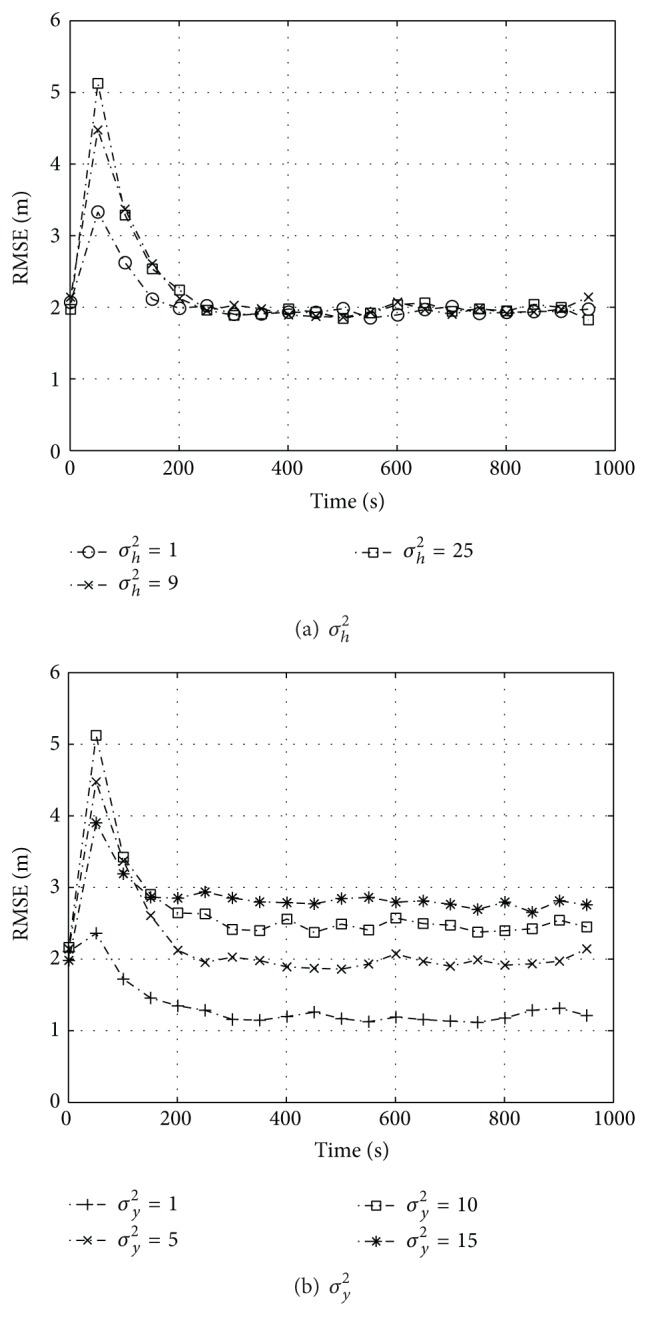
Computer experiments concerning slow fading for RBPF-KF applied to the testbed of Figure 2; positioning RMSE plotted against time for different values of (a) the prior variance of the parameter in the range *σ*_*h*_^2^ = 1 ÷ 25 (*σ*_*y*_^2^ = 5) and (b) the measurement variance *σ*_*y*_^2^ = 1 ÷ 15 (*σ*_*h*_^2^ = 9). The number of particles is *N*_*p*_ = 500 for both.

We also carry out an analysis at different values of *σ*_*h*_^2^ and *σ*_*y*_^2^. In Figure 5 we depict the results concerning RBPF-KF, which show a low sensitivity with regard to *σ*_*h*_^2^ variations in the range [1 ÷ 25]. As for the measurement variance *σ*_*y*_^2^, increasing it by 100% deteriorates the performance by 25%, as it is also reported in terms of steady state values in Table 1.

**Figure 6:**
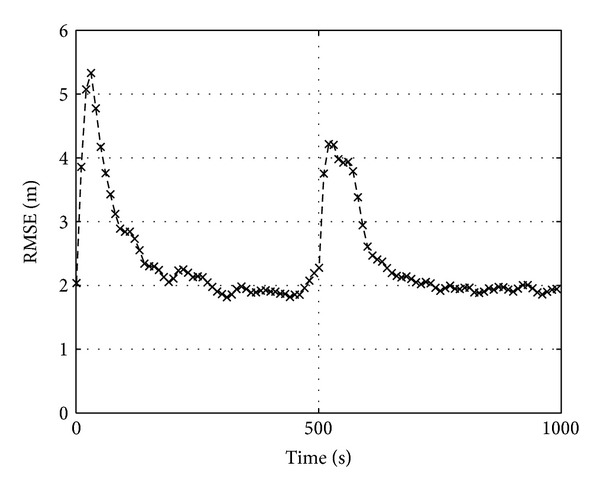
Computer experiments concerning slow fading in the setup of Figure 2 when a −5 dB step variation of the parameter of a single AP at *k* = 500 is imposed in order to mimic a sudden shadowing; here *σ*_*h*_^2^ = 9, *σ*_*y*_^2^ = 5, and *N*_*p*_ = 500.

As a final test, we impose a downside step variation onto the reference power of one AP in order to simulate a sudden obstruction due, for example, to an obstacle. As before, it produces only a further transient in the localization RMSE, but the steady state value keeps unchanged, as shown in Figure 6 for RBPF (see the caption for the simulation details).

### 4.2. Fast Fading (RBPF-AGB)

**Figure 7:**
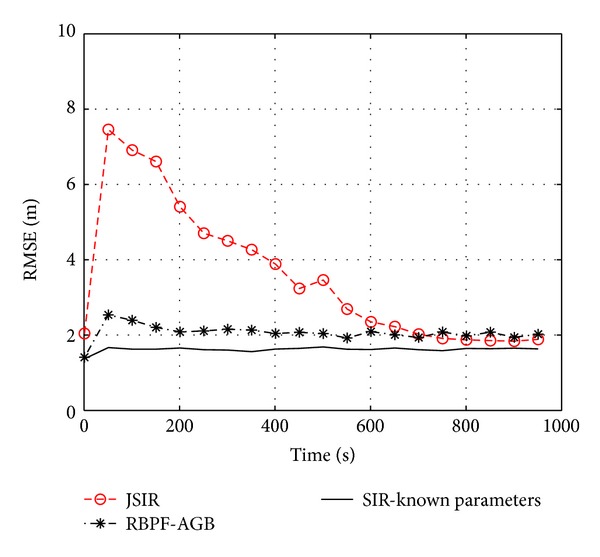
The effect of fast fading on RMSE shown by means of computer experiments on the testbed of Figure 2; JSIR and RBPF-AGB with *δh* = 1 dBm are applied with *σ*_*h*_^2^ = 9, *σ*_*y*_^2^ = 5, and number of particles *N*_*p*_ = 1000.

The fast fading effects are modeled by means of a Rice pdf, as described in Section 2. Thus, we test the RBPF-AGB algorithm, using again the JSIR algorithm as a yardstick.  Figure 7 highlights a comparison between our proposals and the clairvoyant SIR algorithm, applied to the testbed of Figure 2 with *σ*_*h*_^2^ = 9, *σ*_*y*_^2^ = 5, and *N*_*p*_ = 1000. The RBPF-AGB effectiveness is clearly shown, thanks to a very sharp convergence with respect to JSIR, although the steady state value is slightly higher than that of JSIR. This is due to the discrete set of parameter values assumed in RBPF-AGB, whose choice is key for the algorithm performance. We prefer a uniform sampling of **h** in a suitable set, to account for sudden changes during the estimation. The step size, say *δh*, can be tuned by considering the full mismatch case: the maximum difference between the true value of the parameter and the closest discretized value is *δh*/2 and must be lower than the expected error Δ*h*. Since we have found out in the computer experiments that usually Δ*h* ≈ 0.5 dBm, then we choose

(39) $$\begin{array}{c} \delta h=1\;\text{dBm} \end{array}$$

as a suitable balance between algorithm complexity and performance.

**Figure 8:**
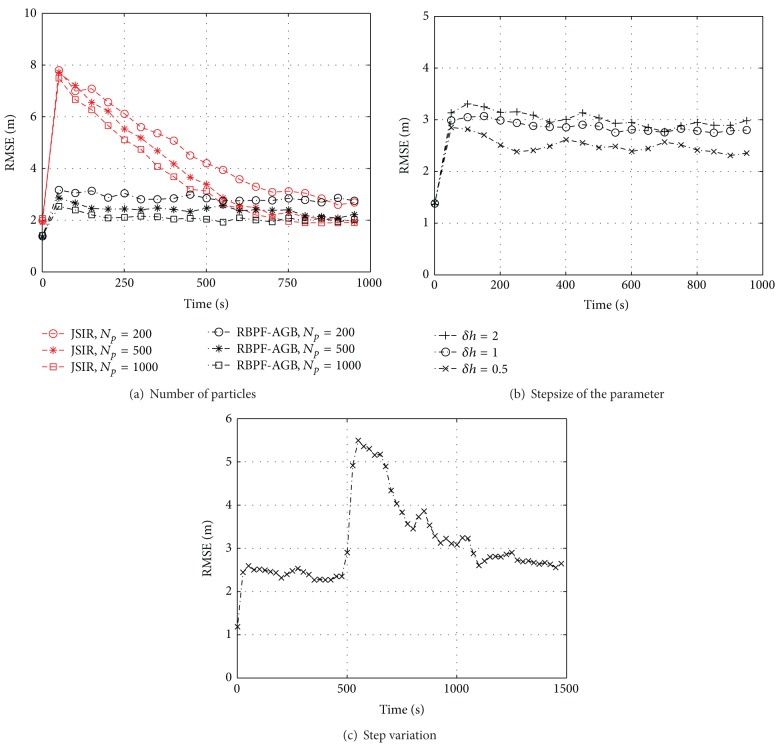
Computer experiments concerning fast fading in the testbed of Figure 2; we report the positioning RMSE plotted against time obtained by (a) both JSIR and RBPF-AGB with different sets of particles in the range *N*_*p*_ = 200 ÷ 1000, (b) RBPF-AGB with step size in the range *δh* = 0.5 ÷ 2, and (c) RBPF-AGB in the presence of a −5 dB step variation on one AP's reference power at the time instant *k* = 500; if not otherwise specified, we use *σ*_*h*_^2^ = 9, *σ*_*y*_^2^ = 5, *N*_*p*_ = 200, and *δh* = 1 dBm.

The results of the analysis relative to the number of particles (*N*_*p*_ = 200 ÷ 1000), step size (*δh* = 0.5 ÷ 2), and downside variation of one AP's reference power are shown in Figure 8, subplots (a), (b), and (c), respectively. In detail, Figure 8(b) confirms that there is room for improvement by setting a lower *δh*. The results about variations of *σ*_*h*_^2^ and *σ*_*y*_^2^ do not present relevant differences compared to the slow fading case. We only report the RMSE steady state values against the measurement variance *σ*_*y*_^2^ in the last column of Table 1.

## 5. Real Data Experiments

**Figure 9:**
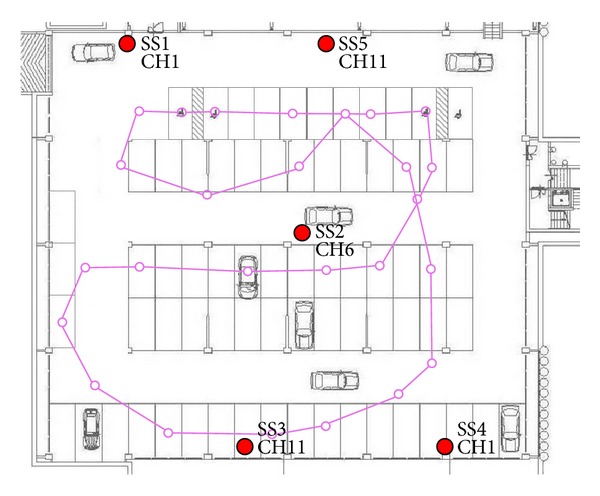
Real world experiments: experimental setting used for data collection. The APs are marked by a red circle and indicated with SS*i*, *i* = 1,…, 5. Also the channel of the 802.11 band used by each AP is indicated. Note that we use only 3 channels in order to avoid interferences, being the distances between SS3 and SS5 and between SS1 and SS4 greater than the APs range in reception. The line in magenta represents the path used in the test and is run several times.

**Figure 10:**
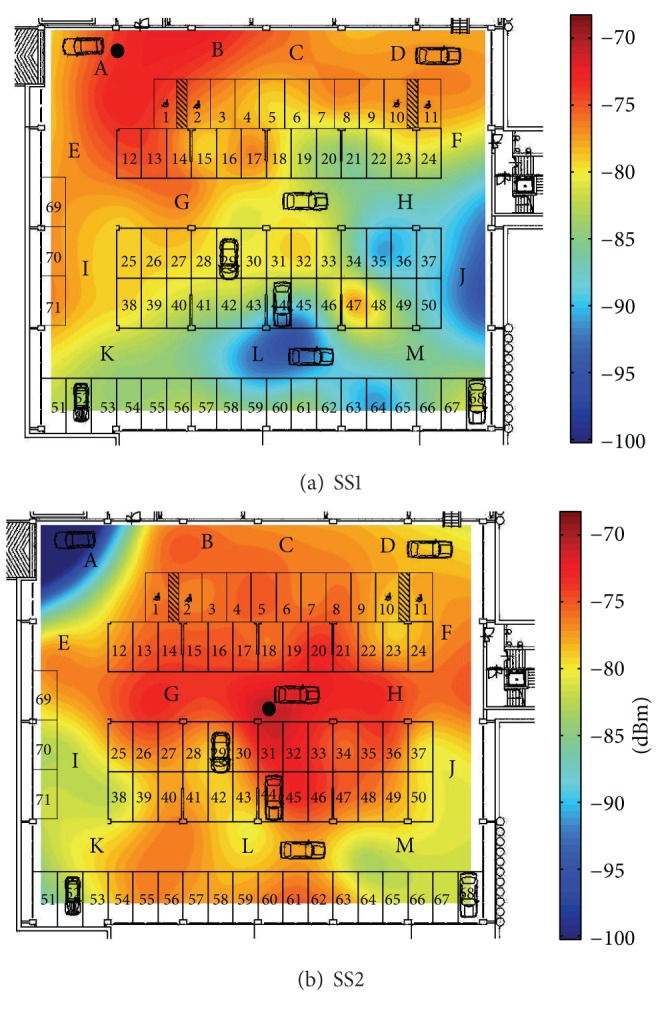
Mean RSS measured in the parking lot related to AP SS1 (a) and AP SS2 (b).

We assess our algorithms on the testbed already presented in [28, 38] and shown in Figure 9. It is a 45 × 40 m indoor parking lot, one floor below the ground level, in which a 802.11 (WiFi) network with 5 APs 3COM 7760 operates. Thick walls, columns made of concrete, and car dispositions which change rapidly make this environment very challenging for indoor localization. That is why in [38] the RADAR algorithm, in its finer weighted version, is shown to exhibit poor performance (sample RMSEs are not lower than 7 meters). In that case the training set, computed on the base of about 30 measurements per 50 positions distributed all over the parking lot, was filled soon before the online stage. In our methods, instead, we estimated in the training stage the decay exponent **α**, the noise variance *σ*_*y*_^2^, and the RSS model. Analysis of measured data reveals an inhomogeneous scenario. As an example, in Figure 10, we show the RSS measured in the target area. The path-loss model defined by (15) is roughly observed with evident fluctuations dependent on the environment configuration. We use for all APs the values *α* = 3 for the decay exponent and *σ*_*y*_^2^ = 20 for the noise variance. We have also observed that the Lognormal model for RSS is dominant and thus we simplify the algorithms by neglecting the fast fading contribution.

**Figure 11:**
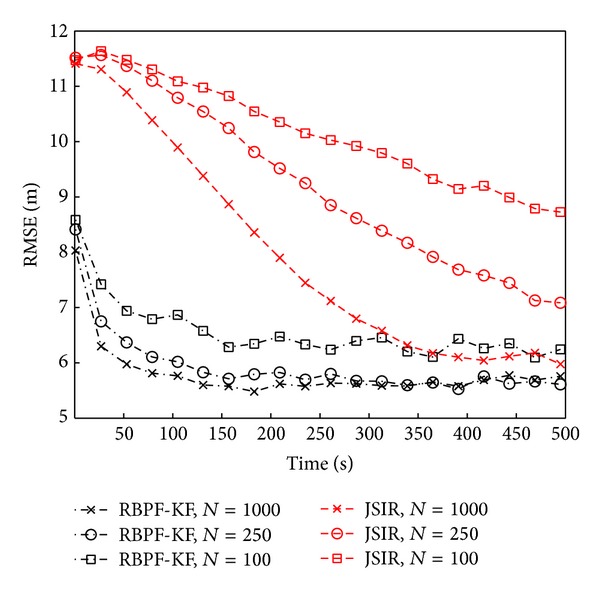
RMSE versus time is represented for RBPF-KF (dashed lines) and JSIR (continuous lines) algorithms applied to real data, with reference to the scenario in Figure 9 for different numbers of particles (*N*_*p*_ = 50 ÷ 1000). We recall that RADAR obtains an RMSE of 7 meters in the same conditions.

The results, presented in Figure 11 for both JSIR and RBPF-KF with different numbers of particles, refer to a 10-minute dataset, acquired along the path shown in Figure 9. They are given in terms of localization RMSE and the ground truth is provided by a set of places known with high accuracy. We can see that both algorithms are convergent to consistent values of the localization RMSE: specifically, RBPF-KF takes less than a minute to achieve errors lower than 6 meters if it is run with at least 250 particles and errors lower than 7 meters if only 100 particles are employed. JSIR is slower, but with 1000 particles its RMSE converges to 6 meters in about 5 minutes.

## 6. Conclusions

Indoor localization employing not perfectly known signals is a challenge still far from a complete solution. We processed RSS measurements, freely available in infrastructured WLANs, by means of an adaptive Bayesian framework which is able to deal with unpredictable effects such as intercalibration and fading. At this aim we referred to simple but very addressed models for signal propagation, whose calibration was carried out online by avoiding time-consuming training stages. Extensive computer experiments and real world data collected in a harsh environment showed the effectiveness of our approaches, evidencing the remarkable convergence properties of the RBPF implementation. A natural continuation of the current work consists in including further propagation parameters within the estimating quantities.

Other future lines of research concern a deeper analysis of the propagation models, aimed at improving the localization accuracy. On the other hand, the development of other tracking techniques that are able to follow other kinds of changes (such as the noise variance) is of paramount importance. A final interesting working case, which will be addressed in the next future, includes the lack of a perfect knowledge of the APs' positions.

## Figures and Tables

**Table 1:** Steady state values of the positioning RMSE computed by means of RBPF algorithm (for both KF and AGB implementations) for different values of the measurement variance in the range σ_*y*_^2^ = 1 ÷ 15. Here σ_*h*_^2^ = 9, *N*_*p*_ = 500, and δ*h* = 1 dBm.

	RBPF-KF	RBPF-AGB
σ_*y*_^2^ = 3	1.6961 m	2.2424 m
σ_*y*_^2^ = 5	1.9600 m	2.8125 m
σ_*y*_^2^ = 10	2.4623 m	2.8657 m
σ_*y*_^2^ = 15	2.7777 m	2.9516 m
